# COGNITIVE FUNCTION AND LONELINESS AMONG OLDER ADULTS: DYNAMICS OF SOCIAL NETWORK AND PERCEIVED SOCIAL SUPPORT

**DOI:** 10.1093/geroni/igac059.2965

**Published:** 2022-12-20

**Authors:** Soobin Park, Sojung Park, BoRin Kim, Jihye Baek, Takashi Amano

**Affiliations:** Washington University in St. Louis, Saint Louis, Missouri, United States; Washington University in Saint Louis, Saint Louis, Missouri, United States; University of New Hampshire, Durham, New Hampshire, United States; Washington University in St. Louis, St. Louis, Missouri, United States; Rutgers University-Newark, Newark, New Jersey, United States

## Abstract

As cognitive reserves diminish with age, older adults become aware of age-related losses and allocate their resources accordingly. Drawing on the selective optimization with compensation and socioemotional selectivity theory, older adults may select emotionally close social relationships to manage emotional distress caused by deterioration in cognitive functions. This study investigates whether different levels of cognitive functions [no impairment (NI), cognitive impairment no dementia (CIND), and dementia] influence the degree of loneliness among older adults, and how heterogeneous patterns of social relations affect this association. We used data from the 2018 Health and Retirement study, which included 4,500 respondents over 51 years of age who completed a modified Telephone Interview for Cognitive Status. Using comprehensive social-relational indicators for the structure and quality of four relational sources (spouse or partner, children, family, and friends), we applied latent class analysis and identified five subgroups of social relations: restricted–limited support (Nf190, 4.22%), family and friends focused–ambivalent (Nf1,252, 27.82%), diverse–positive (Nf2,203, 48.96%), negative (Nf500, 11.11%), and spouse focused–ambivalent (Nf355, 7.89%). Hierarchical regression showed that those in the CIND (p < .032) and dementia group (p < .037) were more likely to experience loneliness than those in the NI group, and those with dementia who were in the category of spouse focused–ambivalent support (p < .001) had relatively lower levels of loneliness. Reliance on spousal support may be due to people selecting and adapting increasingly on closer relations to counter their vulnerability and feel less lonely. Interventions to sustain this core relationship should be encouraged, including spousal-caregiving relief programs.

